# Differential Expression of Three Flavanone 3-Hydroxylase Genes in Grains and Coleoptiles of Wheat

**DOI:** 10.1155/2011/369460

**Published:** 2011-09-29

**Authors:** Eiko Himi, Masahiko Maekawa, Kazuhiko Noda

**Affiliations:** Institute of Plant Science and Resources, Okayama University, Okayama, Kurashiki 710-0046, Japan

## Abstract

Flavonoid pigments are known to accumulate in red grains and coleoptiles of wheat and are synthesized through the flavonoid biosynthetic pathway. Flavanone 3-hydroxylase (F3H) is a key enzyme at a diverging point of the flavonoid pathway leading to production of different pigments: phlobaphene, proanthocyanidin, and anthocyanin. We isolated three *F3H* genes from wheat and examined a relationship between their expression and tissue pigmentation. Three *F3H*s are located on the telomeric region of the long arm of chromosomes 2A, 2B, and 2D, respectively, designated as *F3H-A1*, *F3H-B1*, and *F3H-D1*. The telomeric regions of the long arms of the chromosomes of homoeologous group 2 of wheat showed a syntenic relationship to the telomeric region of the long arm of rice chromosome 4, on which rice *F3H* gene was also located. All three genes were highly activated in the red grains and coleoptiles and appeared to be controlled by flavonoid regulators in each tissue.

## 1. Introduction

Flavonoid pigments are well known to play an important role in pigmentation of tissues such as flowers, fruits, and grains. These pigments not only render the tissues as more conspicuous but also add physiological function to tissues, such as protection against UV damage [1] and increased level of grain dormancy [2].

**Figure 1:**
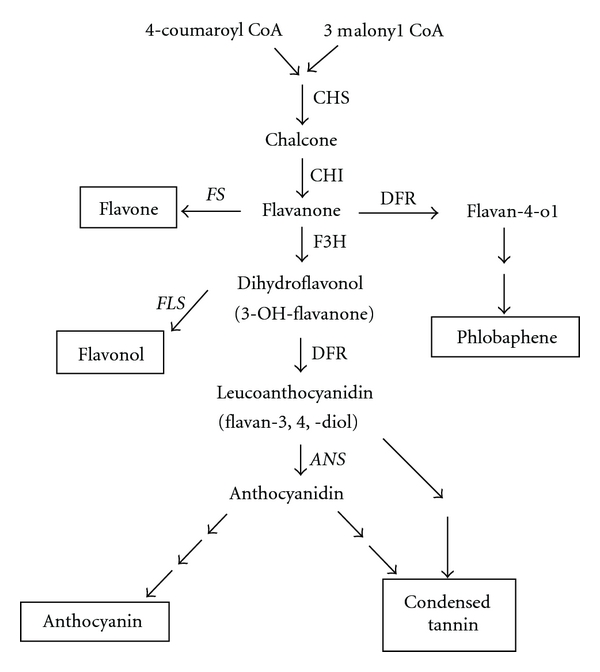
Flavonoid biosynthetic pathway. The *F3H* gene is indicated in bold letters. ANS, FS, and FLS, which belong to 2OG-Fe(II) oxygenase superfamily, are shown in italic. Enzyme names are abbreviated as follows: ANS: anthocyanidin synthase, CHI: chalcone isomerase, CHS: chalcone synthase, DFR: dihydroflavonol 4-reductase, F3H: flavanone 3-hydroxylase, FS: and flavone synthase.

In wheat, red pigmentation was observed in many tissues including grain coats, coleoptiles, anthers, culms, and pericarps. Several genes affecting anthocyanin pigmentation (i.e., *R-1* (*R* in former notation) for red grain, *Rc* for red coleoptile, *Pan* for purple anthers, *Ra *for red auricles, *Pc *for purple culms, and *Pp* for purple pericarp) have been reported [3]. Red-grained wheat has been reported to contain red flavonoid pigments, phlobaphene or proanthocyanidin (condensed tannin), in grain coat tissues [4]. In contrast, pigments of red coleoptiles were anthocyanin [5]. Phlobaphene, proanthocyanidin, and anthocyanin are synthesized through the common flavonoid biosynthetic pathway [6] (Figure 1). Phlobaphenes are compounds produced by polymerization of flavan-4-ols, which are synthesized by three enzymes: chalcone synthase (CHS), chalcone isomerase (CHI), and dihydroflavonol 4-reductase (DFR) in the early steps of the flavonoid pathway. On the other hand, proanthocyanidin and anthocyanin are produced via 3,4-deoxy flavonoids, which are synthesized by four enzymes: CHS, CHI, F3H, and DFR. A step of F3H is a diverging point in the flavonoid pathway leading to the production of either phlobaphene or proanthocyanidin.

Activation of flavonoid biosynthetic genes is required for pigmentation of plant tissue. Transcription factors involved in expression of flavonoid genes have been studied extensively and identified in several plant species, including *Arabidopsis* and maize. The transcription factors, which activate flavonoid genes, are mainly classified into two gene families: one with an MYB domain and the other with a basic helix-loop-helix (bHLH) domain [7]. In maize, C1 (MYB-type) and R (bHLH-type) factors work together leading to the production of anthocyanin. The P (MYB-type) factor of maize alone is responsible for the synthesis of phlobaphene [7]. The TT2 (MYB-type) factor of *Arabidopsis *is required for proanthocyanidin production in the seed coat [8]. Recently, our group showed that *R-1* gene which regulates grain color in wheat was considered to be an MYB-type transcription factor [9].

Transcription factor binding elements have also been studied in promoters of flavonoid biosynthetic genes. Hartmann et al. [10] identified light regulatory units (LRUs) in promoters of *CHS, CHI, F3H*, and flavonol synthase (*FLS*). The LRU consists of two elements: an MYB-recognition element (MRE) and an ACGT-containing element (ACE). Himi and Noda [11] also found a unit of MRE and ACE that was repeated in promoters of wheat *DFR*s.

This paper describes three full sequences of *F3H* genes (*F3H-A1, F3H-B1*, and *F3H*-*D1*), along with their promoters, isolated from hexaploid wheat. These genes were located on the telomeric regions of the long arm of the chromosomes of homoeologous group 2. We also studied a relationship between *F3H* expression and tissue pigmentation of lines with red grain and red coleoptile (*R/Rc*), red grain and white coleoptile (*R/rc*), white grain and red coleoptile (*r/Rc*), and white grain and white coleoptile (*r/rc*). Expressions of *F3H-A1, F3H-B*,*1 *and *F3H-D1* were associated with pigmentation and appeared to be enzymes that are required for pigment synthesis.

## 2. Materials and Methods

### 2.1. Plant Materials

*Triticum aestivum* cvs. Norin 61, Norin 17, Novosibirskaya 67 (NS67), ANK-1C, Chinese Spring (CS), three deletion and three ditelosomic lines of CS were grown under a semitransparent plastic roof in a field (Table 1). The CS spikes were tagged at anthesis and harvested at 5-days postanthesis (DPA). Grains at 5 DPA and the mature stage (water content 15%) were collected from primary and secondary florets of the central spikelets of spikes.

### 2.2. DNA and RNA Extraction

Seedlings were grown at 20°C under darkness for a week and used for DNA preparation. The DNA and RNA were isolated using methods of Himi and Noda [11]. Total RNA was extracted from grains and 3-day-old coleoptiles grown at 20°C under 12 h of UV light (about 100 moL m^−2^ s^−1^, UV lamp) by the sodium dodecyl sulfate-phenol method [11]. Poly(A)^+^ RNA was isolated from 10 mg of the total RNA using an mRNA isolation kit according to the supplier's instructions (Roche Diagnostic Systems Inc., Tokyo, Japan).

### 2.3. Primers Designed for Isolation of Wheat F3H Genes

Primers used in this study were listed in Table 2. Two primers, F3H3LP and F3H2RP, were designed based on the cDNA sequences of barley *F3H* (Accession no. X58138 [12]) and a wheat EST clone, whe24e20 (Accession no. BJ237068) in wheat EST database (http://www.shigen.nig.ac.jp/wheat/komugi/ests/tissueBrowse.jsp), which is similar in nucleotide sequence to barley *F3H*. Partial sequences of wheat *F3H *were amplified using the above primers. New primers were designed in the amplified *F3H* sequences for further amplifications of *F3H* by 3′ RACE, inverse PCR, and RT-PCR.

PCR conditions were as follows: 5 min denaturation at 94°C followed by 30 cycles of 1 min at 94°C, 1 min at 58°C, and 1 min at 72°C, except the RT-PCR and PCR for chromosomal location analysis of *F3H*, in which annealing temperatures used were mentioned later. PCR products were cloned into the pGEM-T vector (Promega Corp., Madison, USA). DNA sequences were determined using the ABI 3100 sequencer (PerkinElmer Inc.) and were analyzed using two software programs: GENETYX (Version 7.0; Software Development, Tokyo, Japan) and CLUSTALW (Bioinformatic Center, Institute for Chemical Research, Kyoto University, available from the web site at http://www.genome.jp/tools/clustalw/).

### 2.4. 3′ RACE and Inverse PCR

**Figure 2:**
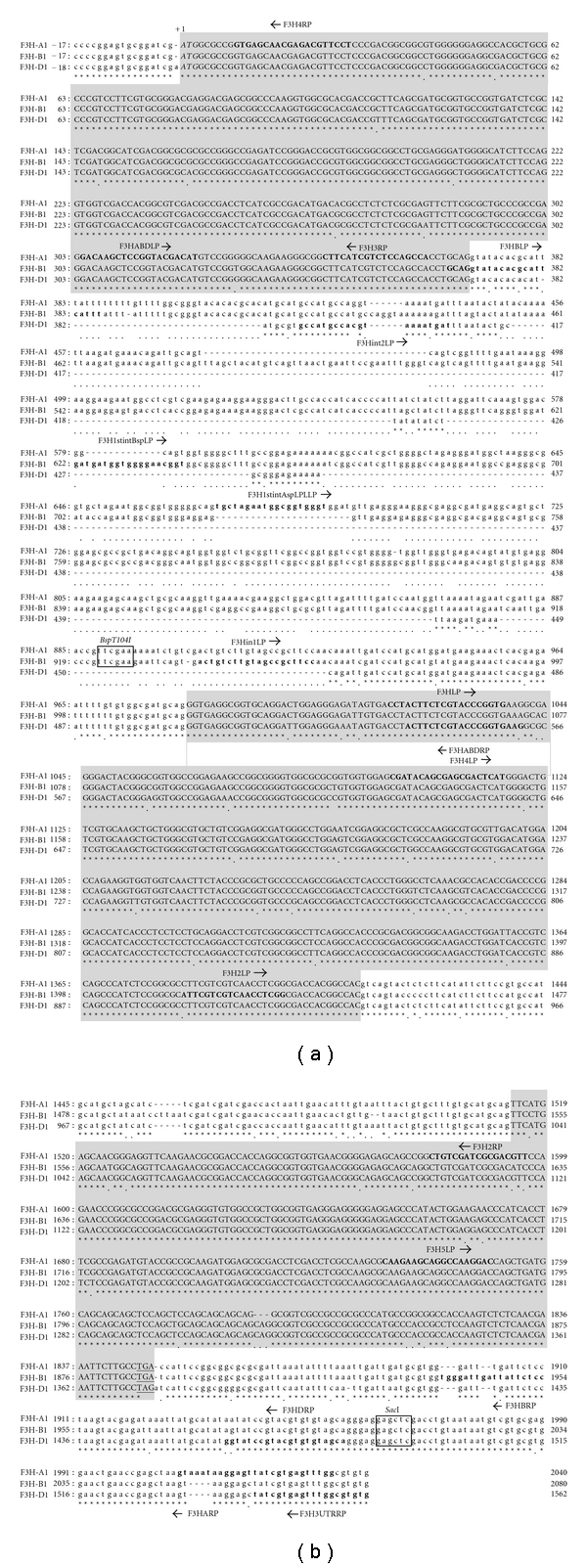
Alignment of the genomic nucleotide sequences of wheat* F3H-A1, F3H-B1, *and* F3H-D1*. Uppercase letters in gray boxes indicate exons: 5′ and 3′ untranslated region and introns are in lowercase letters. Putative translation initiation codon (ATG) is shown in italic and A of ATG is labeled as +1, and stop codons (TGA and TAG) are shown as underlined. Primer sequences are written in bold with the primer name and arrow (right arrow: sense primer; left arrow: antisense primer).

The 3′ regions of *F3H* were amplified in mRNA of grains at 5 DPA of Norin 61 using the 3′ RACE method with F3HLP and F3H2LP primers and an oligo (dT) primer with an adaptor sequence (Table 2, Figure 2).

For inverse PCR, genomic DNA (1.5 *μ*g) of CS was digested with 15 U of one of the following enzymes, *Bsp*T104I (for *F3H-A1*), *Bsp*T104I (for *F3H-B1*), or *Sac* I (for *F3H-D1*) (Figure 2). The DNA was ligated using a ligation high solution (Toyobo Co. Ltd., Japan); the DNA was subsequently used as a template for PCR. The 5′ upstream regions of *F3H* were amplified using inverse PCR method in 20 *μ*L of reaction solution with 30 ng of ligated DNA and 0.5 *μ*M of the primers for inverse PCR listed in Tables 2 and 3. Inverse PCR was carried out first with the first primers and then with the nested primers (2nd and 3rd primers). The transcription factor binding site in the amplified 5′ region was sought using the MOTIF program (Bioinformatic Center, Institute for Chemical Research, Kyoto University, available from the web site at http://motif.genome.ad.jp/).

### 2.5. RT-PCR (Reverse Transcription-PCR)

The first-strand cDNA synthesis and subsequent quantitative RT-PCR assay were carried out according to the method of Himi and Noda [11]. Concentration of cDNA was standardized after evaluating the amount of *actin* mRNA in samples by PCR with the *actin* primers (Table 2). The *F3H-A1* was amplified with F3H5LP and F3HARP primers at annealing temperature of 55°C, *F3H-B1* with F3H5LP and F3HBRP primers at annealing temperature of 60°C, and *F3H-D1* with F3H5LP and F3HDRP primers at annealing temperature of 62°C (Table 3).

### 2.6. Southern Blot Analysis

Genomic DNA of Norin 61 and CS was digested with one of the following restriction enzymes: *Bgl* II, *Eco*R I, *Xba* I, and *Xho* I. Then it was separated on 0.7% (w/v) agarose gel and transferred onto a nylon membrane (Hybond-N+; Amersham Pharmacia Biotech Co. Ltd., Japan). The membranes were prehybridized in a solution of 50% formamide, 5 × SSC (0.75 M NaCl, 75 mM trisodium citrate dihydrate; pH 7.5), 0.1% (w/v) N-lauroylsarcosine, 0.02% SDS, and 2% blocking reagent (Roche Diagnostic Systems Inc.) at 42°C and hybridized for 16 h with a solution containing DIG-labeled probe. The probe was labeled using a PCR DIG Labeling Mix (Roche Diagnostic Systems Inc.) with a pair of primers: F3HLP and F3H2RP (Table 2). The membranes were washed at 65°C with a solution of 0.5 × SSC and 0.1% SDS.

### 2.7. Chromosomal Location

We examined chromosomal locations of *F3H-A1, F3H-B1*, and* F3H-D1* by amplifying these genes in three ditelosomic lines and three deletion lines of CS, which, respectively, lacked the chromosome arm and a part of chromosome (Table 1). Specific primers for *F3H-A1* were F3H1stintAspLP and F3HABDRP. Those for *F3H-B1* were F3H1stintBspLP and F3HABDRP; those for *F3H-D1* were F3Hint2LP and F3H2RP (Table 3). Annealing temperature of 62°C was applied in the PCR to increase specificity of these primers to each *F3H*.

## 3. Results

### 3.1. Copy Number of the F3H Gene

**Figure 3:**
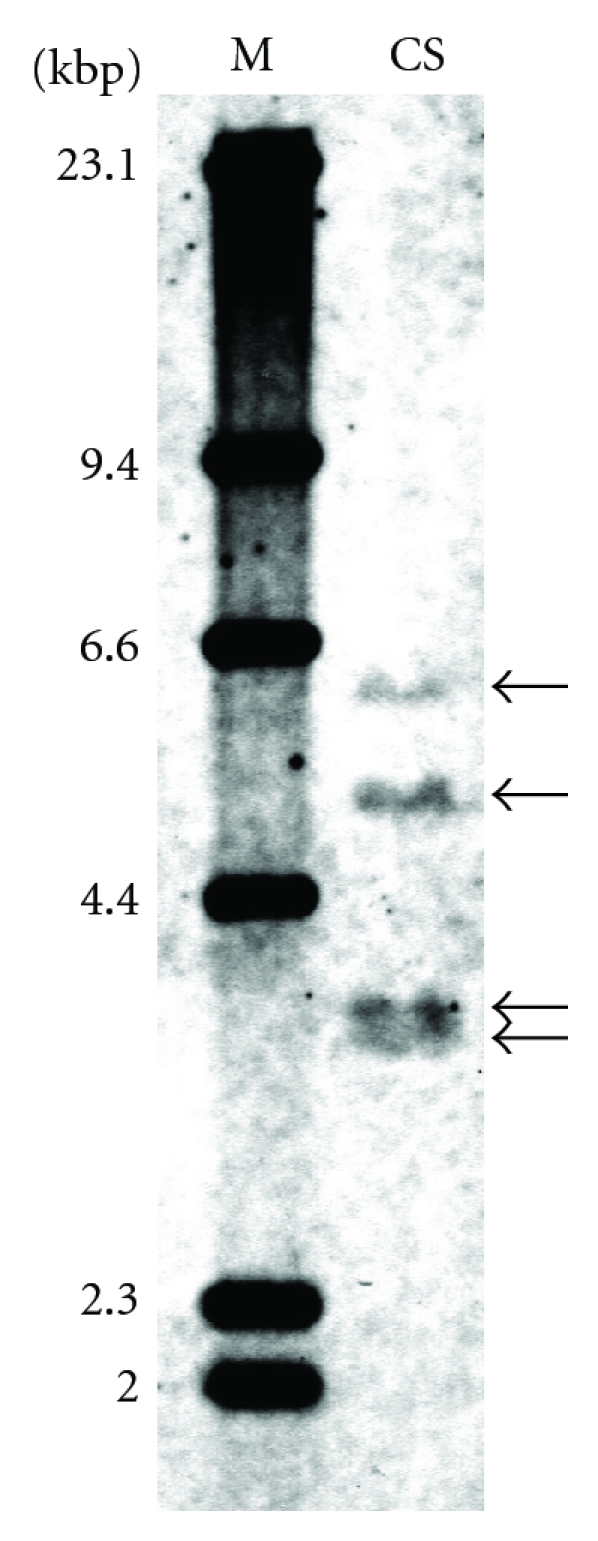
Southern blot of *F3H* gene in genomic DNA of CS digested with an enzyme *Bgl *II. Wheat *F3H* fragment (580-bp) used as a probe was amplified with a pair of F3HLP and F3H2RP primers.

Four fragments of wheat *F3H* genes were detected by Southern blot method in genomic DNA of CS digested with *Bgl* II (Figure 3). Identical numbers of fragments were also observed in Norin 61 DNA digested with *Bgl* II, *Xba* I, or *Xho* I (data not shown). Hexaploid wheat appears to have 4 copies of *F3H* gene.

### 3.2. Identification of F3H-A1, F3H-B1, and F3H-D1

**Figure 4:**
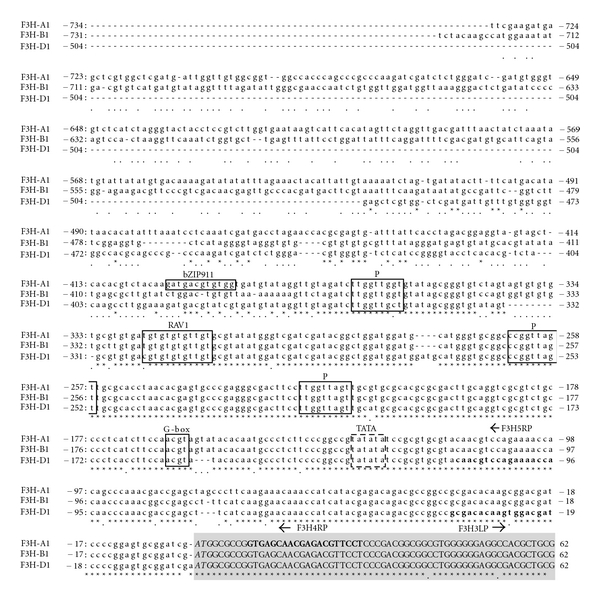
The 5′ region sequences of *F3H-A1, F3H -B1,* and *F3H* -*D1.* Sequences in box indicate the binding sites of transcription factors; the box of broken lines is a putative TATA-box. Exons are indicated in uppercase letters with gray box; the 5′ untranslated region and promoter are in lowercase letters. The putative translation initiation codon (ATG) is shown in italic typeface. Primer sequences are written in bold with primer names and arrows (right arrow: sense primer; left arrow: antisense primer). Elements for transcription factor binding are as follows: bZIP911: bZIP transcription factor of *Antirrhinum majus*; G-box: bZIP-type transcription factor; P: MYB-type transcription factor, P of maize; RAV1: transcription factor with AP2 and B3 domains.

An EST clone of wheat, whe24e20 (Accession no. BJ23706), which is similar in nucleotide sequence to barley *F3H* cDNA (Accession no. X58138 [12]), was found in the integrated wheat science database, KOMUGI (http://www.shigen.nig.ac.jp/wheat/komugi/top/top.jsp), by blast search. The F3H3LP and F3H2RP primers were designed, respectively, in the 5′ UTR near the start codon (ATG) and in the middle region of the *F3H* cDNA sequences (Table 2, Figures 2 and 4). Two PCR products of about 1.6 kbp and 1.2 kbp were obtained in CS genomic DNA using a pair of F3H3LP and F3H2RP primers (see Supplementary Figure  1 in Supplementary Material available online at doi: 10.1155/2011/369460). These products were similar to barley *F3H *and included two putative intron regions. Similarity in the exon region between the 1.6 kbp fragment and barley *F3H* was 94.3%; that between the 1.2 kbp fragment and barley *F3H* was 93.8%.

The 1.2 kbp fragment was not amplified in the ditelo 2DS line in preliminary experiments using 40 ditelosomic lines. This fragment appears to be located on the long arm of chromosome 2D. The results also suggested that the 1.6 kbp fragment was also on the long arms of chromosomes 2A and 2B. We amplified and cloned two 1.6 kbp fragments in ditelo 2AS and 2BS lines, which, respectively, lack the long arms of chromosomes 2A and 2B. The 1.6 kbp fragments amplified in ditelo 2AS and 2BS mutually differed in their nucleotide sequences. These sequences on chromosomes 2AL, 2BL, and 2DL were, respectively, designated tentatively as *F3H-A1, F3H-B1*, and *F3H-D1*.

### 3.3. 3′ Regions of F3H-A1, F3H-B1, and F3H-D1

The 3′ regions of *F3H* amplified by the 3′ RACE included about 200 bp of 3′ UTR. The F3H-3UTRRP primer was designed in the 3′ UTR region (Table 2, Figure 2). The 3′ regions with the second intron were amplified using a pair of primers, F3H2LP and F3H-3UTRRP, in genomic DNA of CS. One amplified fragment was similar in the second intron to *F3H-A1*. The F3Hint1LP and F3Hint2LP primers were newly designed in intron 1 of *F3H-B1* and *F3H-D1* (Table 2, Figure 2). The 3′ regions of *F3H-B1* and *F3H-D1* were amplified with F3Hint1LP and F3H-3UTR primers for *F3H-B1* and F3Hint2LP and F3H-3UTRRP primers for *F3H-D1* (Table 2, Figure 2). The F3HARP primer specific to *F3H-A1*, the F3HBRP primer specific to *F3H-B1*, and the F3HDRP primer specific to *F3H*-*D1 *were designed in the 3′ UTRs (Table 2, Figure 2). These primers were used to amplify cDNA sequences of *F3H*s and to elucidate the expression of each gene.

### 3.4. Nucleotide Sequences after the Start Codon of F3H-A1, F3H-B1, and F3H-D1

**Figure 5:**
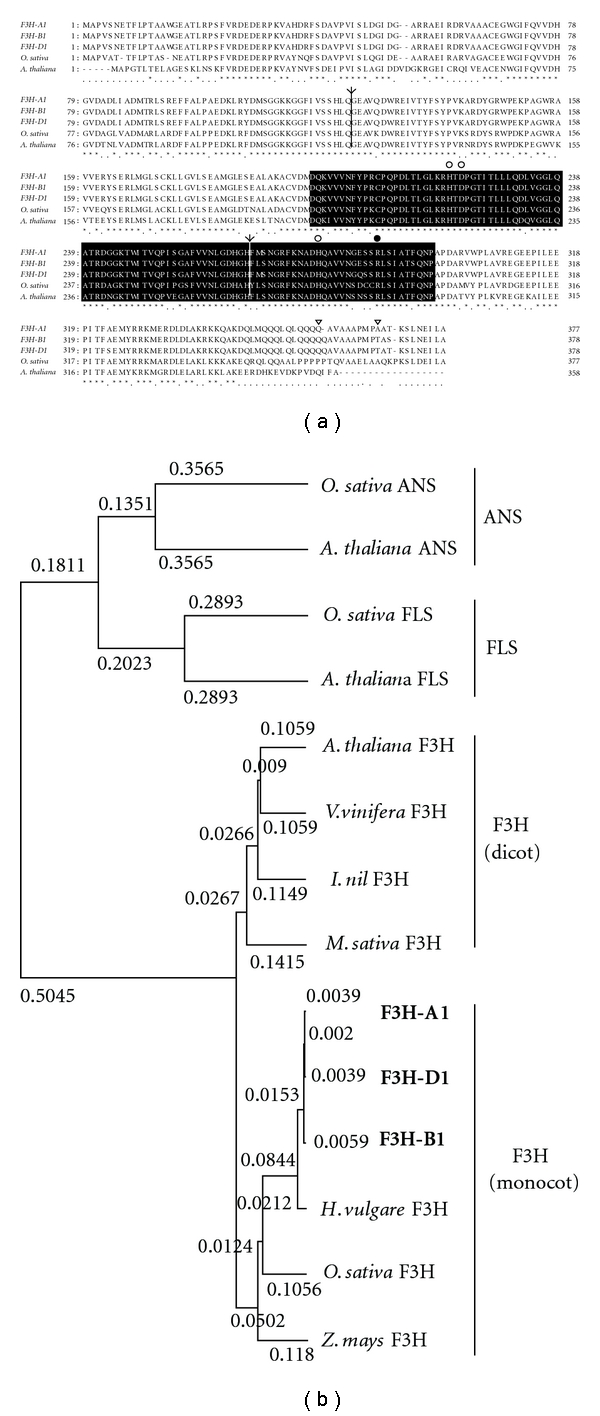
(a) Deduced amino acid sequences of wheat, rice, and *Arabidopsis F3H*. Lines with an arrowhead indicate the intron insertion sites. The region in the black box indicates a characteristic motif, pfam03171, of 2OG-Fe(II) oxygenase superfamily. White circles indicate conserved histidine (H) and aspartic acid (D) residues for ferrous-iron coordination, and a black circle indicates arginine (R) residue for a binding site of 2-oxoglutarate. Triangles show a site of glutamine (Q) residue lacked and an amino acid substitution from threonine (T) to alanine (A) in F3H-A1. (b) UPGMA tree depicted using GENETYX software ver. 7.0, using the following genes belonging to 2OG-Fe(II) oxygenase superfamily: ANSs of *Arabidopsis thaliana *(Q96323) and *Oryza sativa* (CAA69252), FLSs of* Arabidopsis thaliana* (Q96330) and *Oryza sativa* (XP_467968), F3Hs of* Arabidopsis thaliana* (Q9S818), *Hordeum vulgare* (CAA41146), *Ipomea nil* (BAA21897), *Medicago sativa* (S71772), *Oryza sativa* F3H (XP_474226), *Vitis vinifera* (P41090), and *Zea mays* (AAA91227), and F3H-A1 (AB223024), F3H-B1 (AB223025), and F3H-D1 (AB223026) of *Triticum aestivum*.

The full sequences of *F3H-A1, F3H-B1, *and *F3H-D1 *between the start codon and 3′ UTR region were amplified with F3HARP, F3HBRP, F3HDRP, and F3H3LP primers in genomic DNA and cDNAs to examine whether the partial sequences and 3′ regions isolated are in *cis* position (Table 2, Figures 2 and 4). All three *F3H*s had 3 exons and 2 introns; these introns were inserted in the same position as the introns of rice and *Arabidopsis F3H*s (Figure 5(a)). The ORF sequences of wheat *F3H-A1, F3H-B1*, and *F3H-D1 *were similar to each other at more than 96% (*F3H-A1* versus *F3H-B1*: 96.7%, *F3H-A1* versus *F3H-D1*: 96.9%, and *F3H-B1* versus *F3H-D1*: 97.4%), whereas differences exist in sequence and length between the introns, as observed in Figure 2. An F3H enzyme has been reported to have a unique motif of pfam03171 that is maintained commonly in a superfamily of 2-oxoglutarate (2OG) and Fe (II)-dependent oxygenase. Deduced amino acid sequences of wheat *F3H*s also have this pfam03171 motif (Figure 5(a)). Phylogenic relationships among F3Hs of wheat and other plant species were calculated using the UPGMA method of GENETYX software ver. 7.0. Wheat is grouped into the monocotyledon species, including barley, rice, and maize (Figure 5(b)).

### 3.5. Nucleotide Sequences of 5′ Region

The 5′ upstream regions of *F3H-A1, F3H-B1*, and *F3H-D1* were amplified by the inverse PCR. The respective sequences of 734 bp, 731 bp, and 504 bp from the start codon (ATG) of *F3H-A1, F3H-B1*, and *F3H-D1* were isolated and identified as the 5′ regions of respective *F3H* based on the intron sequences of the PCR products (Figure 4). Three P elements to which an MYB-type transcription factor, P, was able to bind were found in these 5′ upstream sequences. A G-box core sequence and an element for RAV1 transcription factor were also found in all three promoters. Only *F3H-A1* had a unique element for a leucine zipper-type transcription factor, bZIP911, in the promoter (Figure 4).

### 3.6. Chromosomal Locations of F3H-A1, F3H-B1, and F3H-D1

**Figure 6:**
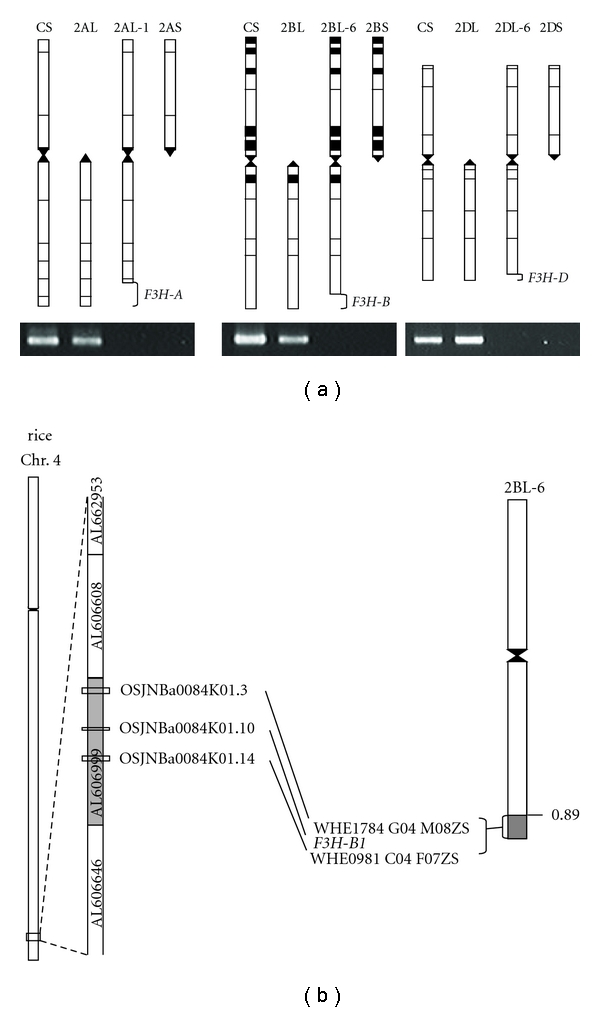
Amplification of *F3H* with the primers specific to *F3H-A1, F3H-B1*, and *F3H-D1* in CS, ditelosomic and deletion lines of chromosomes 2A, 2B, and 2D: (a) amplification of *F3H-A1* in CS, ditelo 2AL, ditelo 2AS, and a partial deletion line of 2AL-1 (FL = 0.85) (left), *F3H-B1* in CS, ditelo 2BL, ditelo 2BS, and a partial deletion line of 2BL-6 (FL = 0.89) (center), and *F3H-D1* in CS, ditelo 2DL, ditelo 2DS, and a partial deletion line of 2DL-6 (FL = 0.94) (right). (b) Locations of two wheat EST clones (WHE1784 G04 M08ZS and WHE0981 C04 F07ZS) and *F3H-B1* on the 0.89–1.00 region of wheat chromosome 2BL, and putative rice *F3H* (OSJNBa0084K01.10) and two rice genes (OSJNBA0084K01.3 and OSJNBa0084K01.14) corresponding to the two wheat ESTs on rice chromosome 4.

Chromosomal locations of *F3H-A1, F3H-B1*, and *F3H-D1* were examined in three deletion lines of CS with primers that were specific to each *F3H* (Tables 2 and 3). No amplification of *F3H-A1, F3H-B1*, and *F3H-D1* was observed in deletion lines 2AL-1 (FL = 0.85), 2BL-6 (FL = 0.89), and 2DL-6 (FL = 0.94), which lack only the small telomeric region of the long arm of chromosomes 2A, 2B, and 2D (Figure 6(a)). These results suggest that *F3H-A1, F3H-B1,* and *F3H-D1* were located, respectively, on the telomeric regions of the long arms of chromosomes 2A, 2B, and 2D. 

### 3.7. Expressions of F3H-A1, F3H-B1, and F3H-D1

**Figure 7:**
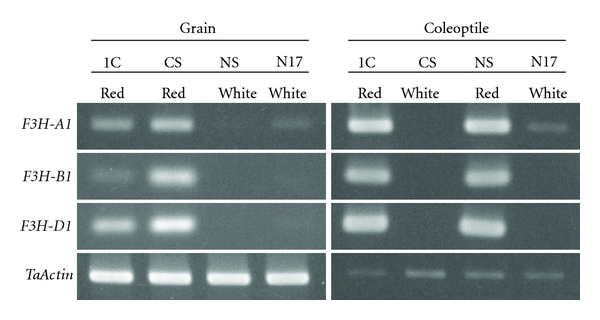
Expression of *F3H-A1, F3H-B1*, and *F3H-D1* in grains and coleoptiles. Respective expressions of *F3H-A1, F3H-B1*, and *F3H-D1* in grains harvested at 5 DPA and 3-day-old coleoptiles were investigated with ANK-1C (red grain and red coleoptile; *R/Rc*), CS (red grain and white coleoptile; *R/rc*), NS67 (white grain and red coleoptile; *r/Rc*), and Norin 17 (white grain and white coleoptile; *r/rc*). *Actin *was used as an internal control.

Respective expressions of *F3H-A1, F3H-B1*, and *F3H-D1* in grains harvested at 5 DPA and 3-day-old coleoptiles were investigated with ANK-1C (red grain and red coleoptile; *R/Rc*), CS (red grain and white coleoptile; *R/rc*), NS67 (white grain and red coleoptile; *r/Rc*), and Norin 17 (white grain and white coleoptile; *r/rc*). F3H5LP and F3HARP primers for *F3H-A1*, F3H5LP and F3HBRP primers for *F3H-B1*, and F3H5LP and F3HDRP primers for *F3H-D1 *were used for RT-PCR (Table 2). All *F3H-A1, F3H-B1,* and *F3H-D1* were highly expressed in red grains of ANK-1C and CS and red coleoptile of ANK-1C and NS67 (Figure 7). On the other hand, no expression of *F3H-A1, F3H-B1*, and *F3H-D1* was detected in white tissues, although *F3H-A1* of grains and coleoptiles of Norin 17 was slightly expressed.

## 4. Discussion

### 4.1. Sequences of Wheat F3H Genes

The deduced amino acid sequences of three wheat *F3H*s were similar to those of *F3H*s of the other monocotyledon species (Figure 5(b)). Three F3Hs of wheat had a characteristic motif (pfam03171) found in the enzymes of 2OG-Fe (II) oxygenase superfamily (Figure 5(a)). Furthermore, 2-oxoglutarate- (2OG-) dependent and Fe (II)-dependent dioxygenases are known to catalyze oxidation of organic substrates using a dioxygen molecule [13]. Flavonol synthase (FLS), anthocyanidin synthase (ANS), and flavone synthase I (FS I) of the flavonoid pathway are also known to have the pfam03171 motif. Two histidines and one aspartic acid of the motif have been identified as sites for putative iron binding; an arginine residue has been identified as a 2-oxoglutarate binding site [14] (Figure 5(a)). These amino acid residues were conserved in wheat F3Hs (Figure 5(a)). Three wheat F3H enzymes appear to function as oxygenases. Recently, two sequences of *F3H* genes on B genome in wheat were isolated and reported as one of these sequences was not detected in red coleoptiles [15]. It is consistent with the result of southern blot which showed four fragments (Figure 3). It appears that the fourth gene *F3H-B2,* a nonhomologous duplication of *F3H-B1,* remains to be characterized.

### 4.2. Chromosomal Location of F3H Genes of Wheat

The results suggest that the *F3H *genes of wheat are located on the telomeric regions of the long arms of chromosomes 2A, 2B, and 2D (Figure 6(a)). Reportedly, there is a syntenic relationship between the chromosomes of wheat homoeologous group 2 and rice chromosomes 4 and 7 [16, 17]. Rice* F3H* (OSJNBa0084K01.10) in BAC clone OSJNBa0084K01 (Accession no. AL606999) was located on the telomeric region of rice chromosome 4 (Figure 6(b)). The BAC clone (OSJNBa0084K01) also included two putative genes, OSJNBa0084K01.3 and OSJNBa0084K01.14, that, respectively, showed high degrees of similarity (more than 80%) to wheat EST clones: WHE1784 G04 M08ZS (Accession no. BF202800) and WHE0981 C04 F07ZS (Accession no. BE500307). These two wheat EST clones were also located on the telomeric region of the long arm of the chromosome 2B (2BL6 0.89–1.00) in the physical bin map of wheat EST clones (http://wheat.pw.usda.gov/wEST/binmaps/) (Figure 6(b)). These results confirmed a high syntenic relationship between the telomeric region of the long arm of the chromosome of wheat homoeologous group 2 and the telomeric region of rice chromosome 4.

### 4.3. Expression of F3H and Pigmentation of Wheat Grain and Coleoptile

*F3H-A1, F3H-B1*, and *F3H*-*D1* were all activated in red grain and coleoptile. On the other hand, only *F3H-A1* of Norin 17 was slightly expressed in white grain and coleoptile. These results suggest that at least *F3H-B1* and *F3H*-*D1 *are involved in pigmentation of the grain and coleoptile. Winkel-Shirley [6] showed that *F3H* took part in the biosynthesis of proanthocyanidin (condensed tannin) and anthocyanins, but not in that of phlobaphene. Pigment synthesized in wheat grain appeared to be proanthocyanidin.

The* F3H-A1* of Norin 17 was expressed in the nonpigmented tissues. Comparison of the deduced amino acid sequence of *F3H-A1* with that of *F3H-B1* and *F3H*-*D1*, *F3H-A1*, showed an amino acid substitution from threonine to alanine and a lack of glutamine residue in the C-terminal region (Figure 5(a)). If these residues play an important role in function, F3H-A1 protein might not be involved in flavonoid biosynthesis. However, this possibility is not probable because these changes in the amino acid sequence occurred far from the characteristic motif, pfam03171, which is a key domain for enzyme function. Himi et al. [5] suggested that *DFR* was not expressed in white grains and coleoptiles and played a critical role in pigment production. Without *DFR* expression, *F3H* expression might not be decisive for pigmentation.

### 4.4. 5′ Region of F3Hs

Three P (MYB-type transcription factor) binding motifs were found in the 5′ region of *F3H-A1, F3H-B1,* and *F3H*-*D1* (Figure 4). Himi and Noda [11] showed that *DFR*s of wheat also had three P motifs in their promoters. The *F3H* and *DFR *of wheat appeared to be controlled by a P-like transcription factor. Recently, we isolated an MYB-type gene of wheat, *Tamyb10*, which might control grain color [9, 18]. It is possible that Tamyb10 protein interacts with the P-binding motifs in promoters of *F3H* and *DFR*.

An RAV1 binding site was also found in the promoters of *F3H-A1, F3H-B1*, and *F3H-D1*. Rice *F3H* (OSJNBa0084K01.10) also had an RAV1 binding site in its promoter. In addition, RAV1 (RAV: for related to ABI3/VP1) was identified as a DNA binding protein possessing an N-terminal AP2/ERF- (or EREBP-) type DNA binding domain and a C-terminal B3 domain [19]; RAV1 is cold-responsive [20]. In *Arabidopsis* and petunia, cold stress (4°C) is known to activate *PAL* and *CHS* expression and to induce the accumulation of anthocyanin in leaves, stems, and flowers [21, 22]. It is likely that an RAV1-like transcription factor also activates wheat *F3H*s because wheat coleoptiles showed more reddish color after chilling.

*F3H-A1* was expressed in white grain and coleoptile. A recognition site of bZIP-type transcription factor (bZIP911) was found only in the promoter of *F3H-A1*. In *Antirrhinum*, bZIP911 reportedly modifies gene expression of histone H4 and chlorophyll a/b (CAB) binding protein [23]. Although no reports have described the bZIP911-like gene in wheat, a bZIP protein such as bZIP911 might activate *F3H-A1* in white grain and coleoptile. Differences in promoter sequences of the *F3H*s increase variation of *F3H* expression and appear to have occurred in evolution during differentiation of three wheat genomes (A, B, and D).

## Figures and Tables

**Table 1:** Wheat lines used in the experiments.

Lines	Grain color	Coleoptile color
Norin 61	Red	White
Norin 17	White	White
Chinese Spring (CS)	Red	White
Deletion lines of CS		
2AL-2	Red	White
2BL-6	Red	White
2DL-6	Red	White
Ditelosomic lines of CS		
ditelo 2AS	Red	White
ditelo 2BS	Red	White
ditelo 2DS	Red	White
Novosibirskaya 67 (NS67)	White	Red
ANK-1C	Red	Red

**Table 2:** Names and sequences of the primers used in the experiments.

Primer	Sequence (5′-3′)	Sequence source	Accession No.
Primers for genomic DNA			
F3H3LP	GCGACACAAGTGGACGAT	whe24e20	BJ237068
F3H2RP	GAACGTCGCGATCGACAG	barley *F3H *(Meldgaard, 1992)	X58138
Primers for 3′ region			
F3HLP	CCTACTTCTCGTACCCGGTG	barley *F3H* (Meldgaard, 1992)	X58138
F3H2LP	ATTCGTCGTCAACCTCGG	barley *F3H* (Meldgaard, 1992)	X58138
Oligo (dT) with 3′ adapter	GGCCACGCGTCGACTAGTACTTTTTTTTTTTTTTTTT		
3′ adapter	GGCCACGCGTCGACTAGTAC		
F3H-3UTRRP	TCTGTCAGACACATGCACACA	3′ region obtained by 3′ RACE	
F3Hint1LP	ACTGTCTTGTAGCCGCTTCC	1st intron of *F3H-A1, B1 *	
Primers for RT-PCR			
F3H5LP	CAAGAAGCAGGCCAAGGAC	3rd exon of *F3H-A1, B1, D1 *	
F3HARP	CCAAACTCACGATAACTCCTTATTTAC	3′ regions of *F3H-A1 *	
F3HBRP	GGAGAATAATCAATCCCACCA	3′ regions of *F3H-B1 *	
F3HDRP	CTGCTACACACGTACGGATACC	3′ regions of *F3H-D1 *	
Primers for inverse PCR and			
chromosomal location analysis			
F3H1stintAspLP	TGCTAGAATGGCGGTGGGT	1st intron of *F3H-A1 *	
F3H1stintBspLP	GATGATGGTGGGGAACGGT	1st intron of *F3H-B1 *	
F3Hint2LP	GCCATGCCACGTAAAATGAT	1st intron of *F3H-D1 *	
F3HABDRP	CTTCACCGGGTACGAGAAGT	2nd exon of *F3H-A1, B1, D1 *	
F3HBLP	GCAGGTATACACGCATTCATTT	1st exon and intron of *F3H-B1 *	
F3H3RP	GTGGCTGGAGACGATGAAG	whe24e20	BJ237068
F3H4LP	CGATACAGCGAGCGACTCAT	2nd exon of *F3H-A1, B1, D1 *	
F3H4RP	AGGAACGTCTCGTTGCTCAC	1st exon of *F3H-A1, B1, D1 *	
F3H5RP	TTGTGGTTTTCTGGACGTTG	5′ regions of* F3H-A1, B1, D1 *	
F3HABDLP	GACAAGCTCCGGTACGACAT	1st exon of* F3H-A1, B1, D1 *	
Primers for *Actin *			
TaActinLP	GAGGGATACACGCTTCCTCA	wheat actin	AB181991
TaActinRP	GAAAGTGCTAAGAGAGGCCAAA	wheat actin	AB181991

**Table 3:** Primers and annealing temperature for each PCR.

	F3H-A1	F3H-B1	F3H-D1	
Location analysis				
Sense	F3H1stintAspLP	F3H1stintBspLP	F3Hint2LP	
Antisense	F3HABDRP	F3HABDRP	F3HABDRP	
Temp.^∗1^	62.0	62.0	62.0	
Expression analysis				
Sense	F3H5LP	F3H5LP	F3H5LP	
Antisense	F3HARP	F3HBRP	F3HDRP	
Temp.^∗1^	55.0	60.0	62.0	
Inverse PCR				
Sense	F3HBLP	F3HABDLP	F3H4LP	1st PCR
Antisense	F3H3RP	F3H4RP	F3HABDRP	
Temp.^∗1^	55.0	55.0	55.0	
Sense	F3H1stintAspLP	F3HBLP	F3H2LP	2nd PCR
Antisense	F3H4RP	F3H5RP	F3H3RP	
Temp.^∗1^	58.0	58.0	58.0	
Sense	F3H1stintAspLP	F3H1stintBspLP	F2H2LP	3rd PCR
Antisense	F3H5RP	F3H5RP	F3H4RP	
Temp.^∗1^	60.0	60.0	60.0	

^∗1^Annealing temperature at PCR reaction.
